# Methods and Tools Used to Estimate the Shortages of Medical Staff in European Countries—Scoping Review

**DOI:** 10.3390/ijerph20042945

**Published:** 2023-02-08

**Authors:** Kamila Parzonka, Costase Ndayishimiye, Alicja Domagała

**Affiliations:** 1 Doctoral School of Medical and Health Sciences, Jagiellonian University Medical College, 31-008 Krakow, Poland; 2 Health Economics and Social Security Department, Institute of Public Health, Faculty of Health Sciences, Jagiellonian University Medical College, 8 Skawińska St., 31-066 Krakow, Poland; 3 Health Policy and Management Department, Institute of Public Health, Faculty of Health Sciences, Jagiellonian University Medical College, 8 Skawińska St., 31-066 Krakow, Poland

**Keywords:** healthcare workforce, staff shortage, health workforce prediction, medical staff deficit, prediction tool, estimation method

## Abstract

Healthcare workforce (HWF) shortages are the biggest challenges today in healthcare systems. Therefore, it is crucial to forecast the future needs of HWFs in order to plan accordingly. The purpose of this study was to identify, map, and synthesize the tools, methods, and procedures for measuring medical staff deficits in Europe. We used the Arksey and O’Malley scoping review methodology. Based on predefined criteria, 38 publications that were retrieved from multiple scientific databases, hand-searched on the internet, from relevant organizations, and scanned from references were considered. They were published between 2002 and 2022. There were 25 empirical studies, 6 theoretical papers, 5 reports, 1 literature review, and 1 guidebook. The majority estimated or measured shortages of physicians (14/38) and nurses (7/38) or looked at HWF generally (10/38). Various methods were used, including projections, estimations, predictions, simulation models, and surveys, which used tools such as special computer software or customized indicators, i.e., the Workload Indicators of Staffing Need method. Researchers estimated HWF shortages at both national and regional levels. Such projections and estimations were often based on demand, supply, and/or need. These methods and tools are not always suited to the needs of a country or medical facility, which is why they need to be further developed and tested.

## 1. Introduction

Globally, healthcare professionals, stakeholders, and politicians involved in the development of health policies and health workforce (HWF) planning are currently facing a variety of challenges [1,2]. Healthcare workforce (HCW) shortages are some of the biggest and most pressing challenges [3]. Despite economic development and technological progress, many countries are still struggling with staff shortages. The trends are worrying as healthcare professionals are aging and there are insufficient efforts to replace retiring professionals. The World Health Organization (WHO) is predicting an increase in the global demand for social and healthcare workers; they estimate that there will be 40 million new health jobs created by 2030 [4,5]. Due to the aging population and the existence of multiple diseases, when assessing shortages, it is necessary to take into account doctors as well as other medical specialists, such as nurses, physiotherapists, pharmacists, and technicians from other medical-related professions, because their work can contribute to reducing the workloads of doctors and ensuring the continuity of care for patients [6].

Organizing adequate care for all patients in need during the COVID-19 pandemic was a particularly huge challenge for healthcare systems throughout the world. The pandemic highlighted the issue of healthcare worker shortages more than ever before. In order to better organize care in similar circumstances in the future, it is necessary to gather information on what resources we have (i.e., the scale of shortages, including the specialists and regions involved) and how they could be used in more optimal ways [7,8].

HWF shortages are conditioned by many factors; however, in order to plan and implement mitigating actions, it is necessary to acquire information on how large these shortages are and what the demands are. Unfortunately, in many countries, there is a lack of strategic planning based on the analysis of the health labor market, exacerbated by the lack of reliable data and information necessary to implement effective human resources policies in healthcare. The collection and subsequent analysis of data usually take time, so the data that are published relate to the previous (or an earlier) year. Therefore, comparing different countries and their shortages may often not be feasible due to the lack of consistent data or the different methods used to assess shortages. It is necessary to reliably measure the sizes of medical staff resources, data on the number of doctors who are licensed and currently working, the number of medical staff who emigrated, and the number of medical workers who have retired. As can be observed, the shortage measurements consist of many factors and their estimations require reliable, thoroughly compiled, and available data. Validated tools and methods are needed to measure, estimate, and prepare shortage forecasts in the near and distant future. They are necessary to plan the admission numbers to medical studies and the number of specialized places, as well as implement policies aimed at mitigating the effects of shortages by applying, for example, task-shifting or solutions using IT [9].

One of the most valuable and comprehensive scientific papers (reviews) on HWF planning is from 2015 [10]. This topic has been studied for many years and was also discussed extensively more than 40 years ago, since 1978 [11]. Many publications and projects have been (and are being) written; however, they tend to cover much broader topics of HWF workforce planning, rather than only focusing on measuring or estimating shortages of medical professionals. Human resource planning for health is extremely important and widely discussed, which is why many scientists attempt to collect the best and most recent data and make them available to interested audiences. Accordingly, literature reviews are available, but they usually cover a select range of methodologies and techniques [12], describe only one group of medical professionals [13], or are limited to a specific time period. Publications have also been published in the following forms: a technical report was published on a specific country [14] and a comprehensive report reviewed 26 OECD projection models used in 18 countries [9]. Global human resource planning strategies [4] and health policy recommendations prepared for the EU are also available [15,16,17]. As mentioned above, many researchers have already addressed the subject of medical workforce planning, as well as considered the possible approaches and available methods, but there is a lack of analysis on health professional shortages in European countries. The main objective of this study was to identify, map, and synthesize the existing publications on estimating or measuring HWF shortages, and the tools, methods, and procedures used for measuring medical staff deficits in European countries. To better explore the topic, we did not apply a publication date limit and included both qualitative and quantitative empirical studies as well as reports, theoretical papers, theses, and gray literature.

## 2. Materials and Methods

The review was performed in accordance with the Arksey and O’Malley [18] scope review methodology and updated by researchers, such as Teare and Taks [19]. The results were analyzed using both qualitative and quantitative methods and presented in accordance with the PRISMA for scoping review checklist (PRISMA-ScR) (Table S1 in Supplementary Materials) developed by Tricco et al. [20]. The final searches were conducted to October 2022. This project was registered with the Open Science Framework (registration DOI: https://doi.org/10.17605/OSF.IO/GKMV7) [21].

### 2.1. Defining Research Questions

The review was guided by the central research question (RQ) as follows: “What are the methods and tools that are used to estimate the shortage of medical staff in Europe?” Other specific questions were derived, most of which reflected the characteristics of the publications and information collected. As a result, the following RQs were created:

RQ1—What kinds of publications and research were carried out?
RQ2—Which countries are included in the study?
RQ3—Which professional groups are covered by the study?
RQ4—What kinds of methods, tools, and procedures were used?
RQ5—Which organizations/stakeholders are involved in predicting HWF shortages?
RQ6—What results and conclusions were stated?
RQ7—What are the research gaps for future studies?

### 2.2. Study Search

The authors searched scientific databases, namely Medline via PubMed, EMBASE, Web of Science, Cochrane Library, as well as manually, by searching for relevant reports. The search strategy combined terms from four themes: (1) method AND (2) estimation AND (3) shortage AND (4) medical staff (Table S2 in Supplementary Materials). Keywords were then derived from these themes and searched by title and/or abstract. The search was not limited to the publication date. Additional studies of interest were identified by visually examining the reference lists of relevant papers. Gray literature was also searched by examining the websites of nine national and international organizations that measure medical staff shortages. Supplementary File—Table S3 presents the operationalization of search strategies used for all databases selected in this scoping review, while Table S4 contains a list of the websites reviewed.

### 2.3. Selection of Publications

The Mendeley Reference Manager was used for deduplication of the retrieved publications. Studies were selected using the Rayyan desktop [22] and consisted of two phases. The first phase was the title and abstract review, and the second one was the full-text review. Two independent researchers, i.e., the authors of this article (K.P. and C.N.), participated in both steps and achieved a sufficiently high level of agreement (98.8%). Therefore, no third investigator was appointed to determine what studies to include. Full-text articles were included according to predefined inclusion and exclusion criteria. They were included if they focused on the tools, methods, models, and procedures used to estimate the HWF shortages in Europe; if they were empirical studies, theoretical works, technical reports, books, chapters, or articles; and if the full text was available in English. They were excluded if they focused on the development of a HWF strategy or human resources for health, i.e., by providing information on the scale of shortages but without any data on estimations or measurements; research on tools and methods used for measuring shortages not related to healthcare workers; came from non-European countries; the publication was the wrong type (conference abstracts, book reviews, commentaries, cover letters, etc.); was in another language; or there was no full text.

### 2.4. Data Extraction

Two data extraction and coding templates were used, i.e., (1) a template for empirical research and (2) a template for other types of research, such as reviews, theoretical papers, guidelines, theses, or reports. Each section in the template corresponds to one research question. However, in the sections, the categories of responses have specific codes assigned to them, which are used in further analyses (in select cases). Data extraction was an iterative process. The researchers independently analyzed 10% of the randomly selected studies, then the results were compared and discussed, and any discrepancies were dispelled during a joint discussion to ensure consistency. The agreement between the two independent investigators was satisfactorily high (98.8% crude agreement); therefore, the data from the remaining publications were screened by only one author.

### 2.5. Gathering and Reporting the Results

The collected data were analyzed using qualitative (thematic) and quantitative techniques. The authors analyzed and classified the included studies according to different categories based on their content and characteristics. First, the descriptive analysis was carried out, including the year of publication, the objective of the study, and the research method used. Secondly, the authors prepared the content analysis, in which they included selected information on models and methods of measuring or estimating medical personnel shortages, such as projection [23], estimations [24], simulations [25], or other quantitative tools [12]. Data were also determined for the group of medical workers included in the study—doctors, dentists, nurses and midwives, physiotherapists, pharmacists, and others. In addition, the research was divided into which international institutions, government organizations, research centers, or other entities were responsible for measuring the shortages of medical staff. The closing part of the methodology provides an overview of the research findings, gaps, limitations, and our conclusion.

## 3. Results

### 3.1. Search Results

**Figure 1:**
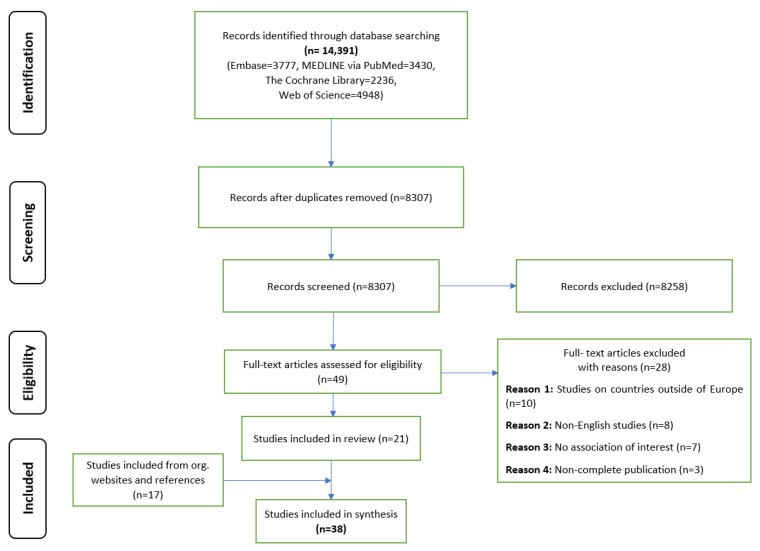
PRISMA Flow Diagram.

We identified 14,391 publications from 4 databases. The Supplementary File (Table S3) shows the results for each database. After removing duplicates, 8307 records were obtained; based on the analysis of the titles and abstracts, 49 full-text articles were included for further analysis. A total of 28 studies were excluded due to their failure to meet the eligibility criteria. After the full-text analysis, 21 articles were classified as meeting the inclusion criteria. An additional 17 articles were included after reviewing the reference lists and conducting a manual search. A total of 38 papers were included in this scoping review, as shown in the PRISMA flowchart (Figure 1). The Supplementary File (Table S5) presents information on the studies considered in the final synthesis.

### 3.2. Characteristics of the Studies Included

**Figure 2:**
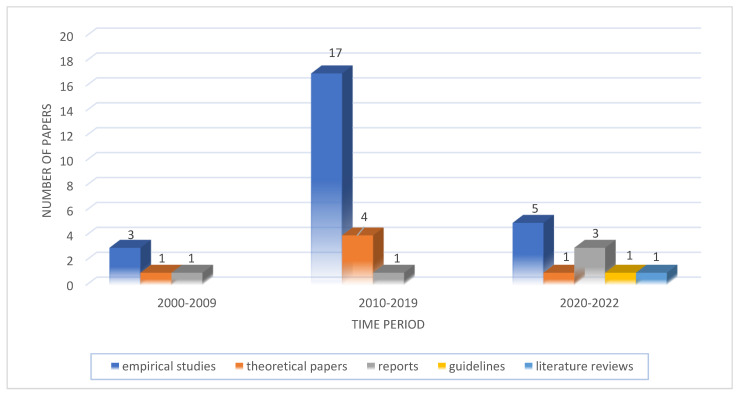
Distribution of studies per type and publication period.

In the 38 publications included, 25 articles [26,27,28,29,30,31,32,33,34,35,36,37,38,39,40,41,42,43,44,45,46,47,48,49,50] were empirical studies. Among the remaining publications, there was one literature review [13], five reports [14,17,51,52,53], six theoretical papers [9,16,54,55,56,57], and one guidebook [15]. Of the 25 empirical studies, approximately 2/3 were quantitative studies, while the remaining ones used mixed research methods. All studies were published between 2002 and 2022. Less than 1/3 of these studies were created more than a decade ago, while around 30% were conducted in the last two years. Detailed data on the number of articles published in individual years are presented in Figure 2. As previously mentioned, other publications included in the review were reports, technical/concept papers, a literature review, and a guidebook. As a result, due to their specificity, a different extraction form was used. The included literature review focuses on nursing resources and how to calculate and counter their shortages [13]. All six reports focus on planning and policies related to healthcare professionals, while one is only concerned with nurses [13]. They identified the drivers of mobility, analyzed the main push-and-pull drivers, and showed how recent movements (and, therefore, shortages) of healthcare workers affect different countries [17,51,52]. One report [14] focused solely on the United Kingdom (England) on the levels of demand and supply, as well as the actions required to ensure access to healthcare services. All of the included reports also considered shortages, mobility, and skill mixes in human resources for health. Only one theoretical paper discussed monitoring and accountability for the implementation of initiatives, such as the EU’s Joint Action on Health Workforce Planning and Forecasting [55], which is related to medical workforce development and planning, as well as demand- and need-based health policies. The third theoretical paper reviewed the most important features and outcomes of 26 HWF projection models (mainly doctors, but also nurses and midwives) in 18 OECD countries [9]. One aimed to contribute to the development of a common pan-European methodology for assessing the shortages of healthcare workers and, consequently, the preparation of legal, political, economic, social, demographic, infrastructural, educational, and administrative instruments for the necessary solution to address the shortages of medical staff [16]. All of the theoretical papers emphasized the importance of having reliable data on human resources for health for monitoring and ensuring accountability for the implementation of regional and national strategies [9,16,54,55,56,57].

### 3.3. Countries Covered in the Included Publications

A total of 5 of the 25 empirical studies were multicountry [35,36,37,49,50], 7 focused on medical staff in the UK [27,38,42,44,45,46,47], and 2 focused on the Netherlands [26,41]. Most studies (6/7) from the UK covered the entire Kingdom [38,46,47] or a select UK state, i.e., England [42,44] and Ireland [45], while 1 study was a case study conducted at the Nuffield Anaesthesiology Unit at John Radcliffe Hospital, Oxford [27]. The remaining eight empirical studies focused on various European countries, such as the Czech Republic [48], Germany [28], Greece [29,40], Lithuania [30,43], Switzerland [31], Spain [32], Malta [33], Hungary [34], and Italy [39], but three focused on select regions of individual countries—the canton of Bern [31], Emilia-Romagna [39], and Hradec Kralove—in comparison with the entire Czech Republic [48]. Two theoretical papers focused on European countries [16,55] and one focused on the OECD states [9]. One literature review focused only on the global shortage of nurses [13]. The reports presented the European perspective on human resources for health, with one report focusing on the EU-28 [17].

### 3.4. Professional Groups Covered by the Publications

**Figure 3:**
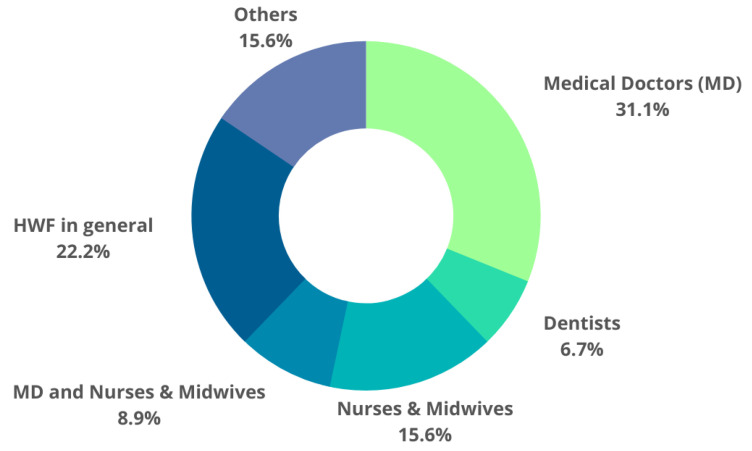
Distribution of professional groups covered by the publications.

A total of 14 of the 38 publications reviewed only concerned medical doctors [26,27,30,31,32,34,36,38,39,43,45,46,49,53]. Many of the papers were about physicians in their selected specialties, mostly general practitioners [14,26,31,34,38,43,45]. We included one study on bowel cancer screening consultants [44], one on vascular surgeons [46], and one that focused on the deficiencies related to anesthetic consultants [27]. Only three studies addressed the shortages of dentists and oral healthcare professionals [41,42] and one addressed the shortage of orthodontists treating children in Greece [29]. Seven papers [13,28,33,40,47,48,56] solely focused on monitoring nurse and/or midwife shortages and/or planning nurse and/or midwife resources. One study focused on registered nurses, a subset of specialized nurses [14], while another compared the work and number of specific types of nurses in the Acute Medicine Unit, i.e., the necessary registered nurses (higher competencies) and non-registered nurses (only allowed to participate in select activities or work, sometimes under supervision) [47]. Only 3 out of 43 papers simultaneously dealt with the shortages of medical doctors, nurses, and midwives (MDs and N&Ms) [14,37,52]. Moreover, we included studies describing healthcare personnel as a whole without identifying specific groups of medical professionals or studies containing doctors, nurses, dentists, pharmacists, and other medical specialists. As shown in the pie chart (Figure 3), publications where the authors targeted more than two professional groups are shown as HWFs in general [9,14,15,16,17,35,50,54,55]. It was only in one study where “others” was associated with a specific group, i.e., trainees [44].

### 3.5. Methods, Tools, and Procedures Applied in the Identified Studies

**Figure 4:**
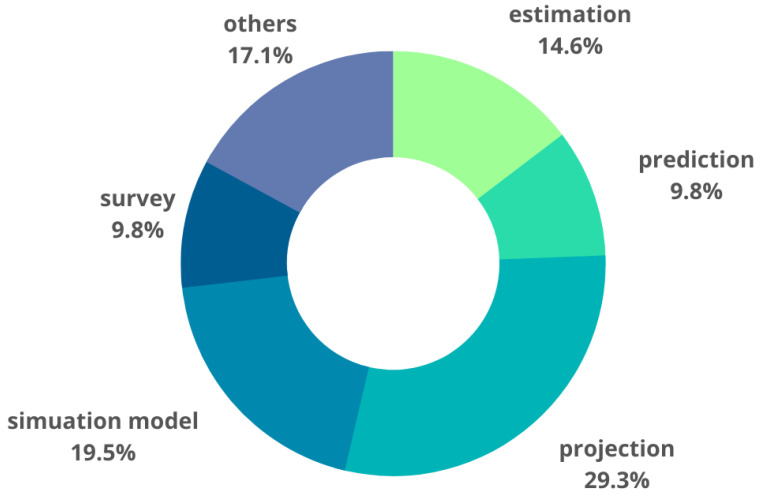
Methods, tools, and procedures identified in empirical studies.

The authors of the included publications used various approaches and methods to obtain information on the demand, supply, and (occurring and forecasted) shortages of medical personnel (see Figure 4). More than 1 method was used in more than half of the studies (18/28) [26,27,30,31,32,34,36,37,38,40,44,45,48,49]. For (almost) every third study in which two methods were used, qualitative and quantitative studies were combined, for example, by conducting a survey before the calculations [30,31,34,44]. Projection was the most common; it was used in more than 1/4 of the included studies [26,28,35,36,39,42,43,44,45,48,50]. In less than every fifth publication, the available medical staff was assessed using mathematical estimations [26,27,31,33,37,47]; equally often (17%), different tools were used that were not assigned to any of the categories listed in Table 1, referred to as “others”. The “others” category includes, inter alia, multiple imputations [38]; interviews [40,48]; multiple stepwise-linear regression used to derive the prediction model and a split-sample cross-validation procedure [49]; the Delphi study [30,32]; association analyses using the deprivation index (DI), i.e., an area-based composite indicator [34]; and usage of the Workload of Indicators Staffing Need (WISN) method [40]. The simulation model was a common projection [32,38,39,42,43,45]. The dynamics system was used in two of the three simulation studies [29,36]; stochastic simulation was used in the third [25], and the Monte Carlo simulation was used in another [39]. A slightly less frequently used method was prediction [27,29,46,49], which accounted for less than 10% of the methods that were presented in the included empirical studies. The definitions of the identified methods, tools, and models are presented in Table 2.

### 3.6. Organizations/Stakeholders Involved in the Process of the Measurement of HWF Shortages

Medical staff shortages have been discussed by various organizations, institutions, politicians, stakeholders, researchers, and employees, i.e., health professionals. International organizations, such as the World Health Organization [35,36,37,50,51], the World Bank [35,37], and the OECD [37] are involved in measuring the demand, supply, and deficits of human resources for health. These organizations also produced data that were used by some researchers in a few of the studies included in our review [35,37,49,50]. The entities involved in the preparation of empirical studies, reports, theoretical papers, guidelines, and reviews on measuring/estimating shortages and medical staff resources were also largely associated with international or national organizations or project teams, including WHO [13,16,35,36,37,50], the Health Foundation’s REAL Centre [14], NHS [14,27], researchers from the EU Public Health Program [55], the Vienna Institute for International Economic Studies [52], the European Commission [17], the World Bank [13,16,35,37], NIVEL [26], and the OECD [16,37]. In some studies, the measurements on medical staff shortages were commissioned, initiated, or supervised by regional/national institutes [28,34,47] or centers [30,45] related to the area of healthcare, the Ministry of Health [32], an observatory [31], researchers [39,40,42,43,46], research associations [29,44], and representatives or workers of a medical institution [27,33,48]. Table 1 presents a complete list of all organizations that participated in the HWF deficiency measurement study.

### 3.7. Results and Conclusions Achieved

Based on the results of the included studies, at least four main methods were used to estimate future needs and, thus, projected shortages of healthcare workers in Europe. First, the ratio of HWF workers to the population seems to be the simplest and, therefore, the most commonly used method. The desired ratio can be determined according to criteria established by various stakeholders, technical agencies, the government, or healthcare workers themselves [51]. Liu et al. [35], in their study, “Global Health Workforce Labor Market Projections for 2030”, used the labor market to forecast future demand for healthcare workers via an economic model, based on health workforce data over 20 years (1990–2013) for 165 countries. The authors compared demand projections with projected growth in the supply of healthcare workers and the requirements of the medical workforce. Based on the results, the researchers predicted that by 2030, there will be an increase in global demand for the healthcare workforce (up to 80 million). As a result, there will be a shortage of 15 million healthcare workers worldwide. With the increasing availability of data related to the medical workforce market, researchers should continually refine future projections of the medical workforce. It would then be possible to simulate alternative models of workforce skill mixes and examine their impacts on service delivery. Moreover, the supply projections do not take into account the outflow rate of the health workforce or the additional number of workers that will need to be trained to replace those leaving the labor market [35].

Second, we look at the utilization (and demand) approach to investigate future requirements based on the information according to levels of the present service utilization, adjusted for future demographic projections [51]. This approach was used to predict a shortage of vascular surgeons in the UK [46]. The researchers considered the number of available vascular surgeons in relation to the UK population; they also considered the number of people per doctor in this specialization, which was 137,000 patients per doctor. The study also included information on the number of people working full-time and part-time, the gender factor, the recommended number of PAs, the number of sessions, and the number of working hours per week. However, the results were deemed unsatisfactory; the surgeons performed more PAs and more sessions, and worked more hours per week than recommended. It was reported that there was a shortage of 275 surgeons [46]. Phenomena, such as shortages or oversupplies of healthcare workers, can be observed at the level of the whole country, but they can also co-exist within a country, with some regions experiencing surpluses and others experiencing shortages at the same time. This poses major economic and political challenges and greatly affects the assessment of the future needs of health professionals. This will motivate researchers to review local, regional, and even single medical facility scales. Information on sick leave, study leave, or maternity leave can be used to estimate or measure HWF shortages. The analysis of local shortages of anesthesiology consultants based on data on leaves in the past and predicted absences made it possible to estimate the demand for services and the resulting shortages in the number of procedures not performed or canceled. Research suggests that a model that factors leave entitlements into the estimation of service capability can accurately predict this real shortfall [47].

Third, the target approach sets targets for the provision and production of specific interventions and transforms them into productivity and staffing standards. It provides insight into the skills and tasks essential to providing specific medical services, allowing for vertical substitution and task-sharing to be taken into account [51].

Finally, the health and service needs approach is used to estimate future HWF needs based on the projected population’s health requirements. This approach defines “service needs” as functions of morbidity trends for a given gender, age, and service norm, and then allows for the recalculation of staffing requirements using performance standards defined by experts [51]. To conduct a needs-based analysis, access to more data is required compared to producing estimates using a supply-based approach. With information from the discussion of a panel of experts, a more accurate simulation model can be prepared to measure the shortage of health professionals; such a technique has been used, among others, in Spain [32] and Lithuania [43]. The simulation model makes it possible to follow the professional life cycles of medical professionals. Many variables and factors affecting the size of the need (depending on the specialization) can be taken into account using advanced computer techniques. An integrated approach is perhaps one of the better ways to address such a complex problem. Integrated approaches can combine a number of indicators and trends, e.g., considering internal reorganization and skill mixes, which were implemented by some researchers in Belgium [53] and Ireland [45].

### 3.8. Research Gaps for Future Studies

The most frequently mentioned limitations in the included empirical studies concerned the availability or quality of data [28,30,31,36,37,38,40,46,48,50], selected methods [26,29,32,34,35,36,37,44,45,47,48,49,50], and specific characteristics of the location where the study was performed, e.g., a single region [31], hospital [33], ward [27], or unit [33,47], which could limit the possibility of generalizing the results obtained. The current states of most country databases are generally inadequate at allowing reliable and valid analyses of the baseline situations. In most of the included studies, the limitations involved preparing analyses, projections, estimates, or models using retrospective data that had been collected in previous years [30,40]. Unfortunately, it allowed planners and researchers to work only with a picture of a situation that had already changed [51]. HWF data are not always comprehensive (often, the informal and private sectors remain black boxes, particularly in social care, where medical staff help as unregistered employees).

Limitations related to the methods focused mainly on the impossibility of a perfect match of the selected tool for assessing medical staff shortages and the many variables affecting them, such as the ages of employees, migration, or work organization. There are information gaps in HWF dimensions, such as activity level and type, migration, multiple types of employment and, in some cases, multiple locations of practice [51]. At times, even if a doctor is registered as an active worker, the doctor is retired and needs this active registration to issue prescriptions or referrals for himself or his family. In addition, definitions of occupational categories vary over time; some professionals have larger scopes of practice, while others have stopped performing some activities, or the same responsibilities (or competencies) are shared by various medical (and even non-medical) specialists, which makes longitudinal comparisons almost impossible. Another major challenge involves using a comprehensive approach to assess needs rather than via individual professions, such as doctors or nurses [52]. This would be needed in a context where various occupational categories are closely related, as indicated by the increasing promotion of task-shifting to help counteract the effects of shortages. In order to properly assess the size of shortages, considering the possession of selected competencies or the possibility of, for example, surgery, it is necessary to agree on what the division and organization of work will look like in the future and how different categories of employees will interact with each other [56]. On the other hand, the gaps indicated by the authors often involved the extrapolation of results in the nursing medical workforce, the different demographic profiles, career options, work, compensation, and status [13]. Even if various tools are used to assess the shortage of healthcare workers, they are not able to take into account the many variables that often occur but are immeasurable, or there is no basis for them to conduct detailed analyses. Of course, these data can be obtained by means of research, e.g., qualitative–in-depth interviews, but unfortunately, they require a lot of time and the involvement of many researchers in the conduct and analysis. In addition, for qualitative research, it is difficult to gather such a large group to obtain information about all possible specialists from many healthcare centers and countries.

## 4. Discussion

### 4.1. Results Summary

The final synthesis included 38 publications, 25 of which were empirical studies, with almost 1/3 published within the last 2 years. There were 5 studies from multiple countries, while the rest were from individual countries, with the UK contributing the most (7/28), as many as all other European countries combined. The most frequently predicted HWF shortages were doctors (14/38), followed by general healthcare workers (10/38), nurses and midwives (7/38), and others (7/38). Such projections and estimations were often based on demand, supply, and/or need. The tools used for estimating the HWFs covered a wide range of innovations, such as mathematical and simulation models. Researchers estimated HWF shortages at both national and regional levels. Additionally, the subject of medical staff shortages was raised by various organizations (e.g., the European Union, the WHO, the OECD, etc.), national health institutions (e.g., ministries of health, etc.), and academic institutions (e.g., universities, etc.), as well as by politicians, stakeholders, independent bodies, relevant professional associations, and individual researchers.

### 4.2. Discussion

As already mentioned, the methods include projections and estimations based on demand, supply, and need. Interestingly, integrated methods and sophisticated tools that enable multifaceted simulations are gaining the most popularity and recognition. In most cases, however, researchers who measure shortages or develop tools to estimate them look at them on a local or regional level. National emphasis was addressed in several reports and theoretical or conceptual papers, such as those from the UK, the Netherlands, Greece, Lithuania, and several other countries. Others focus their research on specific regions, such as the European Union, the WHO regions, or the OECD countries. This reflects the fact that the shortage of healthcare workers is not an isolated local concern within a single country. Instead, it is a concern that is sometimes shared across specific regions. The problem goes even beyond local and regional contexts because the HWF shortage is a preoccupation being addressed by international organizations, such as the WHO, the World Bank, and various multi-center project groups. Medical staff planning has been widely analyzed. We found diverse methods and tools being used to estimate current and future resources and gaps. These tools are prevalent in the context of HWFs in the United States [11,60,61,62,63,64], Canada [65], Australia [66,67], and Europe [51,52,55,57]. On the other hand, no universal–ideal method/approach is available. Despite the increasing progress and development of technology, research-testing current forecasting models shows that there is still a lot to be done, given the differences between the actual results regarding the demand, supply, needs, and shortages and those that were predicted [10,35]. Most interestingly, some of them compared several approaches or combined several tools and methods to obtain the most reliable results. To obtain information on whether there is a shortage or a surplus of medical workers, it is crucial to conduct a gap analysis. This is performed on the basis of a comparison of the supply and existing needs/requirements.

As another example of seeking solutions and continuous development, often fueled by previous criticisms and shortcomings of the supply and needs approach, researchers are constantly developing simulation models [58,68,69]. The use of an open-access simulation tool in Microsoft^®^ Excel (version 2011) has aided medical personnel planning in various countries. Two basic mathematical models are used to quantify the supply and demand of healthcare workers. The supply-side model is based on a stock-and-flow process, and the needs-side model extends the previously published analytical framework using the population health-needs approach. The researchers integrate supply and need analyses, comparing them to identify gaps, both in relative and absolute terms, and then examine their impact on costs for HWF planning strategies and policies. This model was used to simulate a real-life example using midwives and obstetricians/gynecologists in the context of maternal and newborn care in Ghana [68]. The use of simulations and dynamic models also introduces many innovations. Static models enable the estimation of demand and/or supply of the HWF at one time point, while dynamic models [70] provide estimates for various future points in time [71], taking into account the potential and important changes in planning parameters over time. Furthermore, dynamic simulation models allow for the simultaneous analysis of multiple health professions, integrating the planning of more than one group of health professionals into one model so that demand and/or supply estimates for each type of health professional are dependent on others [42].

Even so, forecasting is almost always based on numbers alone. The assessment of future HWF demands extends beyond estimating numbers. Future anticipated needs should be expressed in numerical terms as well as in terms of other aspects of the working environment, such as task shifting and/or skill mix, quality objectives, and productivity. These are critical parameters when creating HWF forecasts and policies [72]. Quantitative tools and models are valuable, but they cannot replace examining and evaluating what is actually needed. As a result, a more thorough approach is required to examine the HWF in its entirety. It is imperative that policymakers understand the expectations and behaviors of healthcare workers and develop strategies to recruit, educate, distribute, retain, motivate, and manage them. Seeing HWF planning as a process would be helpful. Otherwise, it would make little sense to train enough medical professionals, such as nurses and doctors, only to see them leave their homelands when the labor market is not suitable, or the working conditions are not desirable.

In addition, looking at the HWF from different angles and across various medical professions is essential. We found that HWF shortages are often measured and planned on the basis of specific occupations without considering integration within the broader healthcare system or between health professions. Some researchers [2] have raised concerns about not accounting for skill mixes and perceived relationships between healthcare professionals. As healthcare services become more complex and more people seek care, HWFs should be viewed from a variety of health professions [73]. Therefore, researchers [74,75,76,77] proposed using multi-professional aspects of health services. This is justified by the recognized need for teamwork, complementarity among various professional groups of medical professionals, and the nature of interactions occurring in healthcare.

Furthermore, when preparing projections regarding the size of human resources for health, it is important to gather various stakeholders in order to specify strategic goals and the direction of the desired changes. This can also help to identify HWF policy priorities. It may even be helpful for researchers and policymakers to generate consensus on trusted information that can be used as input to a projection model, especially if the available data are unreliable. Choosing a forecasting method or a projective approach requires careful consideration because the type of model used can significantly influence the results and recommendations obtained [51]. Using a needs-based model can produce completely different results than a utility-based model using the same inputs [59]. Some dimensions of the forecasting models are better forecasted at the local level (population needs), while others are better forecasted at the national level (education receipts) as well as at the international level (mobility flows) [55].

Despite more than two decades of work on HWF measurements, existing models continue to oversimplify the complex nature of medical workforce migration and burnout or fail to identify shortages in small or remote locations, because most analyses are country-specific or involve cumulative analyses for many countries at once [54]. Furthermore, there are studies and reports in which the number of shortages is given, but the method of calculating the shortage of the HWF is not provided; therefore, they were not included in the review because they only inform us about the scale of this phenomenon and not the procedure for measuring or estimating it.

### 4.3. Limitations and Implications of the Study

Our study is not free of limitations. The first one is that we included in our analysis only publications that were written in English. The next one is the nature of the methodology of the scoping review and the lack of a quality assessment of the included studies. With the models, methods, and tools identified in our scoping review, it is possible to choose how to identify the shortages of medical staff according to the needs of a given organizational unit, medical facility, or country. Based on this knowledge, it is also possible to compare the approaches presented and implement appropriately tailored health policies aimed at planning and educating a sufficient number of medical workers, thereby ensuring access to medical care, according to the current demand or needs. Based on deeper knowledge of the methods, tools, and models for measuring and estimating the medical staff shortages, decision-makers should use the most effective methods when planning future medical staff, to enable all citizens to have adequate access to high-quality medical services. To this end, stakeholders, politicians, and experts involved in health policy planning and medical management should implement more integrated methods, using simulation models and advanced IT technologies that take into account many variables and important factors at the same time. Constant and close cooperation between stakeholders and medical workers, politicians, and researchers in the fields of epidemiology, statistics, mathematics, computer science, and public health is also necessary in order to explore the current and future needs as extensively as possible and to plan the size of future medical staff in the best possible way. In addition, politicians and decision-makers should strive to ensure that measurements, estimates, and plans, are based only on up-to-date data of the highest quality.

## 5. Conclusions

The results of our review indicate that there are studies that measure or estimate healthcare staff shortages in Europe using a variety of methods and approaches, such as mathematical estimations (including regression models), forecasts, dynamic simulations, or other quantitative and qualitative methods. These methods are not always optimally tailored to the needs of a country, region, or institution, fail to take into account all existing variables, or are based on inaccurate data. Nevertheless, it should be emphasized that thorough healthcare workflow planning requires an approach that is flexible and integrated, taking into account the (potential and effective) demand and supply, as well as other important factors, such as productivity, outcomes, and skill mix. HWF planning is a much broader topic than measuring or estimating the shortages of medical professionals. However, without information about existing shortages and what is foreseen, effective planning would be impossible.

## Figures and Tables

**Table 1:** Empirical studies overview.

Refs.	Author/s and Publication Year	Country	Study Design ^a^	Type of Data ^b^	Profession Group/s ^c^	Methods/ Tools/Procedures Applied	Organizations/Stakeholders Involved in the Process of the Measurement of HWF Shortages
[26]	Van Greuningen et al., 2012	The Netherlands	CS	N/CS	MD	Estimation; Projection	NIVEL; Advisory Committee on Medical Manpower Planning
[27]	Pandit et al., 2010	UK	CS	CS	MD	Estimation; Prediction	NHS; workers at the Nuffield Department of Anaesthetics
[28]	Maier & Afentakis 2013	Germany	CS	N	N	Projection	Federal Institute for Vocational Education and Training (BIBB), The Research Institute of the Federal Employment Agency (IAB)
[29]	Tsiouli et al., 2016	Greece	CS	N	D	Prediction	Greek Association for Orthodontic Study and Research
[30]	Lovkyte et al., 2003	Lithuania	CS	N	MD	Survey; Others	Lithuanian Health Information Centre
[31]	Stierli et al., 2021	Switzerland	CS	R	MD	Survey; Estimation	Swiss Health Observatory (Obsan)
[32]	Barber & López-Valcárcel 2010	Spain	CS	N	MD	Simulation Model	Ministry of Health
[33]	Grech et al., 2012	Malta	LT	N	N	Estimation	Nurse Officers from the Maltese Neonatal Paediatric Intensive Care Unit
[34]	Papp et al., 2019	Hungary	LT	N	MD	Survey; Others	National Public Health Institute, National Institute of Health Insurance Fund Management, Public Health Administration Service of Government Office of Capital City Budapest, MTA-DE Public Health Research Group of the Hungarian Academy of Sciences
[35]	Liu et al., 2017	Multicountry—165 countries	CS	M	MD; N; O	Projection	World Bank; World Health Organization; members of the Global Health Workforce Alliance
[36]	Scheffler et al., 2008	Multicountry—158 countries	CS	M	MD	Projection	World Health Organization
[37]	Scheffler et al., 2018	Multicountry	CS	M	MD; N	Estimation; Projection	World Health Organization, OECD, World Bank
[38]	Taylor et al., 2018	UK	LT	N	MD	Simulation Model; Others	General Medical Council’s
[39]	Lodi et al., 2015	Italy	CS	R	MD	Projection; Simulation Model	Department of Electrical Energy and Information Engineering University of Bologna, Bologna, Italy; Regional Health and Social Agency of Emilia-Romagna, Bologna, Italy; Statistical service and geographical information of the Region Emilia-Romagna, Bologna, Italy
[40]	Gialama et al., 2019	Greece	CS	N	N	Estimation; Others	Department of Social and Educational Policy, University of Peloponnese, Corinth; Department of Nursing, University of Peloponnese, Sparti; Department of Economics, University of Piraeus, Athens; Department of Public Administration, Panteion University, Athens;
[41]	Van Greuningen et al., 2016	The Netherlands	CS	N	D; O	Projection; Others	NIVEL, Dutch Advisory Committee on Medical Manpower Planning
[42]	Gallagher et al., 2010	UK	CS	N	D; O	Projection; Simulation Model	College and St Thomas’ Hospitals, Oral Health Services Research & Dental Public Health London; University of Southampton, Department of Mathematics Operational Research group, Southampton; Cardiff University, WIMCS Operational Research, School of Mathematics, Cardiff
[43]	Starkiene et al., 2005	Lithuania	LT	N	MD	Projection; Simulation Model	Department of Preventive Medicine, Kaunas University of Medicine, Kaunas, Lithuania; Program in Health Services Administration, Xavier University, Ohio, USA
[44]	Ravindran et al., 2021	UK	CS	N	MD; N; O	Survey; Projection	Joint Advisory Group on Gastrointestinal Endoscopy (JAG); the British Society of Gastroenterology (BSG); Association of Coloproctology of Great Britain and Ireland (ACPGBI)
[45]	Teljeur et al., 2010	UK	CS	N	MD	Projection, Simulation Model	Department of Public Health and Primary Care, Trinity College Centre for Health Sciences
[46]	Harkin et al., 2016	UK	CS	N	MD	Prediction	Researchers from the Vascular Surgery United Kingdom Workforce Survey (VSUKWS); National Health Service (NHS);
[47]	Hegarty et al., 2022	UK	CS	N	N	Estimation	National Institute for Health Research, Society of Acute Medicine
[48]	Maresova et al., 2020	Czech Republic	CS	N and R	N	Projection; Others	Healthcare Holding of the Hradec Kralove Region; the Hradec Kralove University Hospital; Hradec Kralove Labour Office
[49]	Tsai et al., 2012	Multicountry—130 countries	CS	M	MD	Prediction; Others	The Health Department in Taiwan
[50]	Boniol et al., 2022	Multicountry	CS	M	MD; N; P; D	Projection	Health Workforce department, World Health Organization

Abbreviation: ^a^  CS—cross-sectional, LT—longitudinal. ^b^ M—multicountry, N—national, R—regional, CS—case study. ^c^ MD—medical doctors, D—Dentists, N—Nurses and midwives, PT—Physiotherapists, P—Pharmacists, O—Others.

**Table 2:** Definitions of identified methods, tools, and models.

Method/Tool/Model.	Definition
Projection	A tool used to better understand future situations and changes (the dynamics of workforce supply and demand) based on a study of current trends and conditions.
Estimation	This process uses data to guess the parameter about the true state of nature.
Prediction	Expectation; this process uses data to guess a random value (variable) that is not part of the dataset.
Simulation model	The process of creating and analyzing a digital prototype of a physical model to predict its performance and various scenarios in the real world.
Survey	The collection of information from a sample of individuals via their responses to questions.
Other methods	
Delphi study	A process of consultations; aims to collect opinions from a group of experts.
Workload of Indicators Staffing Need (WISN) method	A method based on a health worker’s workload, with activity (time) standards applied to each workload component; it is able to calculate the optimal allocation and distribution of staff geographically and functionally between different types of health facilities or health services in a country as a whole.
An area-based composite indicator	A tool built from different indicators; enables the assessment of socioeconomic status (SES) and then allows preparing a comparison between the distribution of unfilled practices and SES.
Interview	A qualitative research method; relies on asking questions in order to collect data.
Multiple stepwise-linear regression	Selection of independent variables to use in a model based on an iterative process of adding or removing variables.
Multiple imputations	A general approach to the problem of missing data that is available in several commonly used statistical packages, which allows repeated imputations to assess the variability of estimated values.

Sources: based on the definition indicated in included studies [23,32,34,38,40,49,58,59].

## Data Availability

Not applicable.

## Supplementary Materials

The following supporting information can be downloaded at: https://www.mdpi.com/article/10.3390/ijerph20042945/s1, Table S1: PRISMA-ScR CHECKLIST; Table S2: Search Terms for the Databases; Table S3: Results of searching selected databases; Table S4: List of organizational websites manually screened; Table S5: List of all included studies.

## References

[1] WHO Regional Office for Europe (2022). Health and Care Workforce in Europe: Time to Act.

[2] Tomblin Murphy G., Birch S., MacKenzie A., Bradish S., Elliott Rose A. (2016). A Synthesis of Recent Analyses of Human Resources for Health Requirements and Labour Market Dynamics in High-Income OECD Countries. Hum. Resour. Health.

[3] Kuhlmann E., Batenburg R., Wismar M., Dussault G., Maier C.B., Glinos I.A., Azzopardi-Muscat N., Bond C., Burau V., Correia T. (2018). A Call for Action to Establish a Research Agenda for Building a Future Health Workforce in Europe. Health Res. Policy Syst..

[4] World Health Organization (2016). Global Strategy on Human Resources for Health: Workforce 2030.

[5] World Health Organization (2016). Working for Health and Growth: Investing in the Health Workforce—Report of the High-Level Commission on Health Employment and Economic Growth.

[6] OECD/European Union (2020). Health at a Glance: Europe 2020: State of Health in the EU Cycle.

[7] da Silva K.R., de Souza F.G., Roquete F.F., da Costa Faria S.M., Peixoto B.C.F., Vieira A. (2020). Allocation of Resources for Health Care in COVID-19 Pandemic Times: Integrative Review. Rev. Bras. Enferm..

[8] Bourgeault I.L., Maier C.B., Dieleman M., Ball J., MacKenzie A., Nancarrow S., Nigenda G., Sidat M. (2020). The COVID-19 Pandemic Presents an Opportunity to Develop More Sustainable Health Workforces. Hum. Resour. Health.

[9] Ono T., Lafortune G., Schoenstein M. (2013). Health Workforce Planning in OECD Countries: A Review of 26 Projection Models from 18 Countries.

[10] Lopes M.A., Almeida Á.S., Almada-Lobo B. (2015). Handling Healthcare Workforce Planning with Care: Where Do We Stand?. Hum. Resour. Health.

[11] Hall T.L., Mejía A., Albul K.V. (1978). Health Manpower Planning: Principles, Methods, Issues.

[12] Subramanian L. (2021). Effective Demand Forecasting in Health Supply Chains: Emerging Trend, Enablers, and Blockers. Logistics.

[13] Drennan V.M., Ross F. (2019). Global Nurse Shortages-the Facts, the Impact and Action for Change. Br. Med. Bull..

[14] Shembavnekar N., Buchan J., Bazeer N., Kelly E., Beech J., Charlesworth A., Mcconkey R., Fisher R. (2022). NHS Workforce Projections 2022.

[15] World Health Organization (2021). Health Labour Market Analysis Guidebook.

[16] Schneider M. (2021). Health Workforce Shortage in EU27 in the Light of Accounting Systems.

[17] Kovács E., Szegner P., Langner L., Sziklai M., Szócska M., Sermeus W., Van Hoegaerden M., Van Deun E., Snyers B. (2021). Mapping of National Health Workforce Planning and Policies in the EU-28.

[18] Arksey H., O’Malley L. (2005). Scoping Studies: Towards a Methodological Framework. Int. J. Soc. Res. Methodol. Theory Pract..

[19] Teare G., Taks M. (2020). Extending the Scoping Review Framework: A Guide for Interdisciplinary Researchers. Int. J. Soc. Res. Methodol..

[20] Tricco A.C., Lillie E., Zarin W., O’Brien K.K., Colquhoun H., Levac D., Moher D., Peters M.D.J., Horsley T., Weeks L. (2018). PRISMA Extension for Scoping Reviews (PRISMA-ScR): Checklist and Explanation. Ann. Intern. Med..

[21] Open Science Framework Open Science Framework Registries. https://osf.io/gkmv7.

[22] Johnson N., Phillips M. (2018). Rayyan for Systematic Reviews. J. Electron. Resour. Librariansh..

[23] Kirch D.G., Henderson M.K., Dill M.J. (2012). Physician Workforce Projections in an Era of Health Care Reform. Annu. Rev. Med..

[24] Morgan P. (2019). Predicted Shortages of Physicians Might Even Disappear If We Fully Account for PAs and NPs. J. Am. Acad. PAs.

[25] Pando-Ezcurra T., Auccahuasi W., Saenz Arenas E.R., Rosario Pacahuala E.A., González Ponce de León E.R., Olaya Cotera S., Flores Castañeda R.O., Herrera L. (2022). Method for the Analysis of Health Personnel Availability in a Pandemic Crisis Scenario through Monte Carlo Simulation. Appl. Sci..

[26] Van Greuningen M., Batenburg R.S., Van der Velden L.F. (2012). Ten Years of Health Workforce Planning in the Netherlands: A Tentative Evaluation of GP Planning as an Example. Hum. Resour. Health.

[27] Pandit J.J., Tavare A.N., Millard P. (2010). Why Are There Local Shortfalls in Anaesthesia Consultant Staffing?: A Case Study of Operational Workforce Planning. J. Health Organ. Manag..

[28] Maier T., Afentakis A. (2013). Forecasting Supply and Demand in Nursing Professions: Impacts of Occupational Flexibility and Employment Structure in Germany. Hum. Resour. Health.

[29] Tsiouli K., Karamesinis K., Antonarakis G.S., Christou P. (2016). Prediction Model of Regional Orthodontic Workforce Needs, Using Greece as an Example. Eur. J. Paediatr. Dent..

[30] Lovkyte L., Reamy J., Padaiga Z. (2003). Physicians Resources in Lithuania: Change Comes Slowly. Croat. Med. J..

[31] Stierli R., Rozsnyai Z., Felber R., Jörg R., Kraft E., Exadaktylos A.K., Streit S. (2021). Primary Care Physician Workforce 2020 to 2025—A Cross-Sectional Study for the Canton of Bern. Swiss Med. Wkly..

[32] Barber P., López-Valcárcel B.G. (2010). Forecasting the Need for Medical Specialists in Spain: Application of a System Dynamics Model. Hum. Resour. Health.

[33] Grech V., Cassar M., Distefano S. (2012). Nurse Staffing Levels on the NPICU in the Island of Malta. J. Pediatr. Intensive Care.

[34] Papp M., Korosi L., Sandor J., Nagy C., Juhasz A., Adany R. (2019). Workforce Crisis in Primary Healthcare Worldwide: Hungarian Example in a Longitudinal Follow-up Study. BMJ Open.

[35] Liu J.X., Goryakin Y., Maeda A., Bruckner T., Scheffler R. (2017). Global Health Workforce Labor Market Projections for 2030. Hum. Resour. Health.

[36] Scheffler R.M., Liu J.X., Kinfu Y., Dal Poz M.R. (2008). Forecasting the Global Shortage of Physicians: An Economic- and Needs-Based Approach. Bull. World Health Organ..

[37] Scheffler R.M., Campbell J., Cometto G., Maeda A., Liu J., Bruckner T.A., Arnold D.R., Evans T. (2018). Forecasting Imbalances in the Global Health Labor Market and Devising Policy Responses. Hum. Resour. Health.

[38] Taylor C., McManus I.C., Davison I. (2018). Would Changing the Selection Process for GP Trainees Stem the Workforce Crisis? A Cohort Study Using Multiple-Imputation and Simulation. BMC Med. Educ..

[39] Lodi A., Tubertini P., Grilli R., Mazzocchetti A., Ruozi C., Senese F. (2016). Needs Forecast and Fund Allocation of Medical Specialty Positions in Emilia-Romagna (Italy) by System Dynamics and Integer Programming. Health Syst..

[40] Gialama F., Saridi M., Prezerakos P., Pollalis Y., Contiades X., Souliotis K. (2019). The Implementation Process of the Workload Indicators Staffing Need (WISN) Method by WHO in Determining Midwifery Staff Requirements in Greek Hospitals. Eur. J. Midwifery.

[41] Van Greuningen M. (2016). Health Workforce Planning in the Netherlands.

[42] Gallagher J.E., Kleinman E.R., Harper P.R. (2010). Modelling Workforce Skill-Mix: How Can Dental Professionals Meet the Needs and Demands of Older People in England?. Br. Dent. J..

[43] Starkiene L., Smigelskas K., Padaiga Z., Reamy J. (2005). The Future Prospects of Lithuanian Family Physicians: A 10-Year Forecasting Study. BMC Fam. Pract..

[44] Ravindran S., Munday J., Veitch A.M., Broughton R., Thomas-Gibson S., Penman I.D., McKinlay A., Fearnhead N.S., Coleman M., Logan R. (2022). Bowel Cancer Screening Workforce Survey: Developing the Endoscopy Workforce for 2025 and Beyond. Frontline Gastroenterol..

[45] Teljeur C., Thomas S., O’Kelly F.D., O’Dowd T. (2010). General Practitioner Workforce Planning: Assessment of Four Policy Directions. BMC Health Serv. Res..

[46] Harkin D.W., Beard J.D., Shearman C.P., Wyatt M.G., Surg R.C. (2016). Predicted Shortage of Vascular Surgeons in the United Kingdom: A Matter for Debate?. Surgeon.

[47] Hegarty H., Knight T., Atkin C., Kelly T., Subbe C., Lasserson D., Holland M. (2022). Nurse Staffing Levels within Acute Care: Results of a National Day of Care Survey. BMC Health Serv. Res..

[48] Maresova P., Prochazka M., Barakovic S., Baraković Husić J., Kuca K. (2020). A Shortage in the Number of Nurses-A Case Study from a Selected Region in the Czech Republic and International Context. Healthcare.

[49] Tsai T.-C., Eliasziw M., Chen D.-F. (2012). Predicting the Demand of Physician Workforce: An International Model Based on “Crowd Behaviors”. BMC Health Serv. Res..

[50] Boniol M., Kunjumen T., Nair T.S., Siyam A., Campbell J., Diallo K. (2022). The Global Health Workforce Stock and Distribution in 2020 and 2030: A Threat to Equity and “universal” Health Coverage?. BMJ Glob. Health.

[51] Dussault G., Buchan J., Sermeus W., Padaiga Z. (2010). Assessing Future Health Workforce Needs.

[52] Mara I. (2020). Health Professionals Wanted: Chain Mobility across European Countries.

[53] Roberfroid D., Stordeur S., Camberlin C., Van de Voorde C., Vrijens F., Leonard C. (2008). Physician Workforce Supply in Belgium: Current Situation and Challenges.

[54] World Health Organization (2010). Models and Tools for Health Workforce Planning and Projections.

[55] Kroezen M., Van Hoegaerden M., Batenburg R. (2018). The Joint Action on Health Workforce Planning and Forecasting: Results of a European Programme to Improve Health Workforce Policies. Health Policy.

[56] Simoens S., Villeneuve M., Hurst J. (2005). Tackling Nurse Shortages in OECD Countries.

[57] Malgieri A., Michelutti P., Van Hoegaerden M. (2015). Handbook on Health Workforce Planning Methodologies across EU Countries.

[58] MacKenzie A., Tomblin Murphy G., Audas R. (2019). A Dynamic, Multi-Professional, Needs-Based Simulation Model to Inform Human Resources for Health Planning. Hum. Resour. Health.

[59] Hurst K., Ford J., Keen J., Mottram S., Robinson M. (2002). Selecting and Applying Methods for Estimating the Size and Mix of Nursing Teams: A Systematic Review of Literature Commissioned by Department of Health.

[60] Lakhan S.E., Laird C. (2009). Addressing the Primary Care Physician Shortage in an Evolving Medical Workforce. Int. Arch. Med..

[61] Birch S., Kephart G., Tomblin-Murphy G., O’Brien-Pallas L., Alder R., MacKenzie A. (2007). Human Resources Planning and the Production of Health: A Needs-Based Analytical Framework. Can. Public Policy.

[62] Birch S., Kephart G., Murphy G.T., O’Brien-Pallas L., Alder R., MacKenzie A. (2009). Health Human Resources Planning and the Production of Health: Development of an Extended Analytical Framework for Needs-Based Health Human Resources Planning. J. Public Health Manag. Pract..

[63] Goodman D.C., Fisher E.S., Bubolz T.A., Mohr J.E., Poage J.F., Wennberg J.E. (1996). Benchmarking the US Physician Workforce: An Alternative to Needs-Based or Demand-Based Planning. JAMA.

[64] Sloan F.A. (1977). Access to Medical Care and the Local Supply of Physicians. Med. Care.

[65] Lomas J., Stoddart G.L., Barer M.L. (1985). Supply Projections as Planning: A Critical Review of Forecasting Net Physician Requirements in Canada. Soc. Sci. Med..

[66] Joyce C.M., McNeil J.J., Stoelwinder J.U. (2004). Time for a New Approach to Medical Workforce Planning. Med. J. Aust..

[67] Duckett S.J. (2005). Health Workforce Design for the 21st Century. Aust. Health Rev..

[68] Asamani J.A., Christmals C.D., Reitsma G.M. (2021). Advancing the Population Needs-Based Health Workforce Planning Methodology: A Simulation Tool for Country Application. Int. J. Environ. Res. Public Health.

[69] Manzi S., Chalk D., Day J., Pearson M., Lang I., Stein K., Pitt M. (2018). A Novel Modelling and Simulation Capacity Development Initiative for the National Health Service. BMJ Simul. Technol. Enhanc. Learn..

[70] Tomblin Murphy G., MacKenzie A., Guy-Walker J., Walker C. (2014). Needs-Based Human Resources for Health Planning in Jamaica: Using Simulation Modelling to Inform Policy Options for Pharmacists in the Public Sector. Hum. Resour. Health.

[71] US Department of Health and Human Services (2017). Supply and Demand Projections of the Nursing Workforce: 2014–2030.

[72] Dreesch N., Dolea C., Dal Poz M.R., Goubarev A., Adams O., Aregawi M., Bergstrom K., Fogstad H., Sheratt D., Linkins J. (2005). An Approach to Estimating Human Resource Requirements to Achieve the Millennium Development Goals. Health Policy Plan..

[73] International Labour Organization Securing Decent Work for Nursing Personnel and Domestic Workers, Key Actors in the Care Economy. Proceedings of the International Labour Conference, 110th Session.

[74] Jones R., Bhanbhro S.M., Grant R., Hood R. (2013). The Definition and Deployment of Differential Core Professional Competencies and Characteristics in Multiprofessional Health and Social Care Teams. Health Soc. Care Community.

[75] Klaasen K., Bowman S., Komenda P. (2016). Advancing Interprofessional Collaborative Teams in the Winnipeg Health Region. Healthc. Q..

[76] Curson J.A., Dell M.E., Wilson R.A., Bosworth D.L., Baldauf B. (2010). Who Does Workforce Planning Well? Workforce Review Team Rapid Review Summary. Int. J. Health Care Qual. Assur..

[77] Masnick K., McDonnell G. (2010). A Model Linking Clinical Workforce Skill Mix Planning to Health and Health Care Dynamics. Hum. Resour. Health.

