# Supplementary material for: Methods and Tools Used to Estimate the Shortages of Medical Staff in European Countries—Scoping Review
Source: Int J Environ Res Public Health. 2023 Feb 8;20(4):2945. doi: 10.3390/ijerph20042945 (PMC9957245; doi:10.3390/ijerph20042945)
Supplement: Supplementary file 1 [file ijerph-20-02945-s001.zip › ijerph-2157398-supplementary.pdf]

Table S1. PRISMA-ScR CHECKLIST

SECTION	ITEM	PRISMA-ScR CHECKLIST ITEM	REPORTED ON PAGE #
**TITLE**			
Title	1	Identify the report as a scoping review.	1
**ABSTRACT**			
Structured summary	2	Provide a structured summary that includes (as applicable): background, objectives, eligibility criteria, sources of evidence, charting methods, results, and conclusions that relate to the review questions and objectives.	1
**INTRODUCTION**			
Rationale	3	Describe the rationale for the review in the context of what is already known. Explain why the review questions/objectives lend themselves to a scoping review approach.	2-3
Objectives	4	Provide an explicit statement of the questions and objectives being addressed with reference to their key elements (e.g., population or participants, concepts, and context) or other relevant key elements used to conceptualize the review questions and/or objectives.	3
**METHODS**			
Protocol and registration	5	Indicate whether a review protocol exists; state if and where it can be accessed (e.g., a Web address); and if available, provide registration information, including the registration number.	3
Eligibility criteria	6	Specify characteristics of the sources of evidence used as eligibility criteria (e.g., years considered, language, and publication status), and provide a rationale.	4
Information sources*	7	Describe all information sources in the search (e.g., databases with dates of coverage and contact with authors to identify additional sources), as well as the date the most recent search was executed.	3
Search	8	Present the full electronic search strategy for at least 1 database, including any limits used, such that it could be repeated.	3 (Supplementary file, Table S3 listed on page 3)
Selection of sources of evidence†	9	State the process for selecting sources of evidence (i.e., screening and eligibility) included in the scoping review.	4
Data charting process‡	10	Describe the methods of charting data from the included sources of evidence (e.g., calibrated forms or forms that have been tested by the team before their use, and whether data charting was done independently or in duplicate) and	4

		any processes for obtaining and confirming data from investigators.	
Data items	11	List and define all variables for which data were sought and any assumptions and simplifications made.	4
Critical appraisal of individual sources of evidence§	12	If done, provide a rationale for conducting a critical appraisal of included sources of evidence; describe the methods used and how this information was used in any data synthesis (if appropriate).	n/a
Synthesis of results	13	Describe the methods of handling and summarizing the data that were charted.	4
**RESULTS**			
Selection of sources of evidence	14	Give numbers of sources of evidence screened, assessed for eligibility, and included in the review, with reasons for exclusions at each stage, ideally using a flow diagram.	5
Characteristics of sources of evidence	15	For each source of evidence, present characteristics for which data were charted and provide the citations.	6-8
Critical appraisal within sources of evidence	16	If done, present data on critical appraisal of included sources of evidence (see item 12).	n/a
Results of individual sources of evidence	17	For each included source of evidence, present the relevant data that were charted that relate to the review questions and objectives.	6-12
Synthesis of results	18	Summarize and/or present the charting results as they relate to the review questions and objectives.	4-16
**DISCUSSION**			
Summary of evidence	19	Summarize the main results (including an overview of concepts, themes, and types of evidence available), link to the review questions and objectives, and consider the relevance to key groups.	15-17
Limitations	20	Discuss the limitations of the scoping review process.	17
Conclusions	21	Provide a general interpretation of the results with respect to the review questions and objectives, as well as potential implications and/or next steps.	17
**FUNDING**			
Funding	22	Describe sources of funding for the included sources of evidence, as well as sources of funding for the scoping review. Describe the role of the funders of the scoping review.	n/a

JBI = Joanna Briggs Institute; PRISMA-ScR = Preferred Reporting Items for Systematic reviews and Meta-Analyses extension for Scoping Reviews.

* Where *sources of evidence* (see second footnote) are compiled from, such as bibliographic databases, social media platforms, and Web sites.

† A more inclusive/heterogeneous term used to account for the different types of evidence or data sources (e.g., quantitative and/or qualitative research, expert opinion, and policy documents) that may be eligible in a scoping review as opposed to only studies. This is not to be confused with *information sources* (see first footnote).

‡ The frameworks by Arksey and O'Malley (6) and Levac and colleagues (7) and the JBI guidance (4, 5) refer to the process of data extraction in a scoping review as data charting.

§ The process of systematically examining research evidence to assess its validity, results, and relevance before using it to inform a decision. This term is used for items 12 and 19 instead of "risk of bias" (which is more applicable to systematic reviews of interventions) to include and acknowledge the various sources of evidence that may be used in a scoping review (e.g., quantitative and/or qualitative research, expert opinion, and policy document).
From: Tricco AC, Lillie E, Zarin W, O'Brien KK, Colquhoun H, Levac D, et al. PRISMA Extension for Scoping Reviews

Table S2. Search Terms for the Databases

**Topic**	**Search terms**
Method	method* OR tool* OR model*
Estimation	estimat* OR predict* OR plan* OR forecast* OR measure*
Shortage	shortag* OR gap* OR shortfall* OR maldistribution* OR deficit*
Medical staff	"medical staff* OR physician* OR doctor* OR nurse* OR workforce* OR "health professional*" OR "health worker*"

Table S3: Results of searching selected databases

Embase Search Strategy		
Database: Embase		
Steps:<br>• Step1: #1<br>• Step2: #2<br>• Step3: #3<br>• Step4: #4<br>• Step5: #1 AND #2 AND #3 AND #4<br>• Step6: filters (English language, article, human studies)		
#1	method*:ab,ti OR tool*:ab,ti OR model*:ab,ti	14,659,239 Results
#2	estimat*:ab,ti OR predict*:ab,ti OR plan*:ab,ti OR forecast*:ab,ti OR measure*:ab,ti	9,317,831 Results
#3	shortag*:ab,ti OR gap*:ab,ti OR shortfall*:ab,ti OR maldistribution*:ab,ti OR deficit*:ab,ti	777,075 Results
#4	'medical staff*':ab,ti OR physician*:ab,ti OR doctor*:ab,ti OR nurse*:ab,ti OR workforce*:ab,ti OR 'health professional*':ab,ti OR 'health worker*':ab,ti	1,262,151 Results
#5	((((method*:ab,ti OR tool*:ab,ti OR model*:ab,ti) AND estimat*:ab,ti OR predict*:ab,ti OR plan*:ab,ti OR forecast*:ab,ti OR measure*:ab,ti) AND shortag*:ab,ti OR gap*:ab,ti OR shortfall*:ab,ti OR maldistribution*:ab,ti OR deficit*:ab,ti) AND 'medical staff*':ab,ti OR physician*:ab,ti OR doctor*:ab,ti OR nurse*:ab,ti OR workforce*:ab,ti OR 'health professional*':ab,ti OR 'health worker*':ab,ti)	11,589 Results
#6	((((method*:ab,ti OR tool*:ab,ti OR model*:ab,ti) AND estimat*:ab,ti OR predict*:ab,ti OR plan*:ab,ti OR forecast*:ab,ti OR measure*:ab,ti) AND shortag*:ab,ti OR gap*:ab,ti OR shortfall*:ab,ti OR maldistribution*:ab,ti OR deficit*:ab,ti) AND 'medical staff*':ab,ti OR physician*:ab,ti OR doctor*:ab,ti OR nurse*:ab,ti OR workforce*:ab,ti OR 'health professional*':ab,ti OR 'health worker*':ab,ti) AND [article]/lim AND [english]/lim AND [humans]/lim	3,777 Results

<table>
<tr><td colspan="3">PubMed Search strategy</td></tr>
<tr><td colspan="3">Database: MEDLINE via PubMed</td></tr>
<tr><td colspan="3">Steps:
• Step1: #1
• Step2: #2
• Step3: #3
• Step4: #4
• Step5: #1 AND #2 AND #3 AND #4
• Step6: filters (Full text, English language)</td></tr>
<tr><td>#1</td><td>Search: method*[Title/Abstract] OR tool*[Title/Abstract] OR model*[Title/Abstract]</td><td>10,508,638 Results</td></tr>
<tr><td>#2</td><td>Search: estimat*[Title/Abstract] OR predict*[Title/Abstract] OR plan*[Title/Abstract] OR forecast*[Title/Abstract] OR measure*[Title/Abstract]</td><td>7,293,301 Results</td></tr>
<tr><td>#3</td><td>Search: shortage*[Title/Abstract] OR gap*[Title/Abstract] OR shortfall*[Title/Abstract] OR maldistribution*[Title/Abstract] OR deficit*[Title/Abstract]</td><td>520,856 Results</td></tr>
<tr><td>#4</td><td>Search: “medical staff*”[Title/Abstract] OR physician*[Title/Abstract] OR doctor*[Title/Abstract] OR nurse*[Title/Abstract] OR workforce*[Title/Abstract] OR “health professional*”[Title/Abstract] OR “health worker*”[Title/Abstract]</td><td>941,684 Results</td></tr>
<tr><td>#5</td><td>Search: (((method*[Title/Abstract] OR tool*[Title/Abstract] OR model*[Title/Abstract]) AND (estimat*[Title/Abstract] OR predict*[Title/Abstract] OR plan*[Title/Abstract] OR forecast*[Title/Abstract] OR measure*[Title/Abstract])) AND (shortage*[Title/Abstract] OR gap*[Title/Abstract] OR shortfall*[Title/Abstract] OR maldistribution*[Title/Abstract] OR deficit*[Title/Abstract])) AND (“medical staff*”[Title/Abstract] OR physician*[Title/Abstract] OR doctor*[Title/Abstract] OR nurse*[Title/Abstract] OR workforce*[Title/Abstract] OR “health professional*”[Title/Abstract] OR “health worker*”[Title/Abstract])</td><td>6,589 Results</td></tr>
<tr><td>#6</td><td>Search: (((method*[Title/Abstract] OR tool*[Title/Abstract] OR model*[Title/Abstract]) AND (estimat*[Title/Abstract] OR predict*[Title/Abstract] OR plan*[Title/Abstract] OR forecast*[Title/Abstract] OR measure*[Title/Abstract])) AND (shortage*[Title/Abstract] OR gap*[Title/Abstract] OR shortfall*[Title/Abstract] OR maldistribution*[Title/Abstract] OR deficit*[Title/Abstract])) AND (“medical staff*”[Title/Abstract] OR physician*[Title/Abstract] OR doctor*[Title/Abstract] OR nurse*[Title/Abstract] OR workforce*[Title/Abstract] OR “health professional*”[Title/Abstract] OR “health worker*”[Title/Abstract]) Filters: Full text, English</td><td>3,430 Results</td></tr>
</table>

Cochrane Search Strategy		
Database: The Cochrane Library		
Steps:<br>• Step1: #1<br>• Step2: #2<br>• Step3: #3<br>• Step4: #4<br>• Step5: #1 AND #2 AND #3 AND #4<br>• Step6: filters (Cochrane Reviews, New Search)		
#1	Search: method* OR tool* OR model* in Record Title OR method* OR tool* OR model* in Abstract	8,576 Results
#2	Search: estimat* OR predict* OR plan* OR forecast* OR measure* in Record Title OR estimat* OR predict* OR plan* OR forecast* OR measure* in Abstract	5,908 Results
#3	Search: shortag* OR gap* OR shortfall* OR maldistribution* OR deficit* in Record Title OR shortag* OR gap* OR shortfall* OR maldistribution* OR deficit* in Abstract	388 Results
#4	Search: medical staff* OR physician* OR doctor* OR nurse* OR workforce* OR health professional* OR health worker* in Record Title OR medical staff* OR physician* OR doctor* OR nurse* OR workforce* OR health professional* OR health worker* in Abstract	1481 Results
#5	Search: method* OR tool* OR model* AND estimat* OR predict* OR plan* OR forecast* OR measure* AND shortag* OR gap* OR shortfall* OR maldistribution* OR deficit* AND medical staff* OR physician* OR doctor* OR nurse* OR workforce* OR health professional* OR health worker* in Record Title OR method* OR tool* OR model* AND estimat* OR predict* OR plan* OR forecast* OR measure* AND shortag* OR gap* OR shortfall* OR maldistribution* OR deficit* AND medical staff* OR physician* OR doctor* OR nurse* OR workforce* OR health professional* OR health worker* in Abstract	8,591 Results
#6	Search: method* OR tool* OR model* AND estimat* OR predict* OR plan* OR forecast* OR measure* AND shortag* OR gap* OR shortfall* OR maldistribution* OR deficit* AND medical staff* OR physician* OR doctor* OR nurse* OR workforce* OR health professional* OR health worker* in Record Title OR method* OR tool* OR model* AND estimat* OR predict* OR plan* OR forecast* OR measure* AND shortag* OR gap* OR shortfall* OR maldistribution* OR deficit* AND medical staff* OR physician* OR doctor* OR nurse* OR workforce* OR health professional* OR health worker* in Abstract - in Cochrane Reviews	2,236 Results

Web of Science Search Strategy
Database: Web of Science Core Collection
Steps:

• Step1: #1<br>• Step2: #2<br>• Step3: #3<br>• Step4: #4<br>• Step5: #1 AND #2 AND #3 AND #4<br>• Step6: filters (Full text, English language)		
#1	method* OR tool* OR model* (Abstract) or method* OR tool* OR model* (Title)	16,051,233 Results
#2	estimat* OR predict* OR plan* OR forecast* OR measure* (Abstract) or estimat* OR predict* OR plan* OR forecast* OR measure (Title)	12,169,572 Results
#3	shortag* OR gap* OR shortfall* OR maldistribution* OR deficit* (Abstract) or shortag* OR gap* OR shortfall* OR maldistribution* OR deficit* (Title)	932,873 Results
#4	"medical staff* OR physician* OR doctor* OR nurse* OR workforce* OR "health professional*" OR "health worker*" (Abstract) or "medical staff* OR physician* OR doctor* OR nurse* OR workforce* OR "health professional*" OR "health worker*" (Title)	478,003 Results
#5	(method* OR tool* OR model*) AND (estimat* OR predict* OR plan* OR forecast* OR measure*) AND (shortag* OR gap* OR shortfall* OR maldistribution* OR deficit*) AND ("medical staff* OR physician* OR doctor* OR nurse* OR workforce* OR "health professional*" OR "health worker*") (Abstract) or (method* OR tool* OR model*) AND (estimat* OR predict* OR plan* OR forecast* OR measure*) AND (shortag* OR gap* OR shortfall* OR maldistribution* OR deficit*) AND ("medical staff* OR physician* OR doctor* OR nurse* OR workforce* OR "health professional*" OR "health worker*") (Title)	5,891 Results
#6	(method* OR tool* OR model*) AND (estimat* OR predict* OR plan* OR forecast* OR measure*) AND (shortag* OR gap* OR shortfall* OR maldistribution* OR deficit*) AND ("medical staff* OR physician* OR doctor* OR nurse* OR workforce* OR "health professional*" OR "health worker*") (Abstract) or (method* OR tool* OR model*) AND (estimat* OR predict* OR plan* OR forecast* OR measure*) AND (shortag* OR gap* OR shortfall* OR maldistribution* OR deficit*) AND ("medical staff* OR physician* OR doctor* OR nurse* OR workforce* OR "health professional*" OR "health worker*" (Title) and Articles (Document Types) and English (Languages)	4,948 Results

Table S4. List of organizational websites manually screened

№	Organization	Website	Date of the search
1	World Health Organization	https://www.who.int/teams/health-workforce	20.07.2022

2	European Union	https://european-union.europa.eu/index_en	22.09.2022
3	Global Health Workforce Alliance	https://www.who.int/teams/health-workforce/workforcealliance	20.07.2022
4	Joint Action on European Health Workforce Planning and Forecasting (EU JAHWF)	https://healthworkforce.eu/	20.07.2022
5	European Institute of Health and Sustainable Development	https://eihsd.eu/trends-of-health-workforce-migration-in-the-european-union-2/	22.09.2022
6	Netherlands Institute for Health Services Research (NIVEL)	https://www.nivel.nl/en/publicatie/health-workforce-planning-netherlands-how-projection-model-informs-policy-regarding	20.07.2022
7	The Vienna Institute for International Economic Studies	https://wiiw.ac.at/health-professionals-wanted-chain-mobility-across-european-countries-p-5339.html	20.07.2022
8	The Health Foundation	https://www.health.org.uk/publications/nhs-workforce-projections-2022	22.09.2022
9	The Belgian Health Care Knowledge Centre	https://www.kce.fgov.be/	22.09.2022

Table S5: List of all included studies

**List of included studies**				
**No.**	**Reference number**	**First author/s and publication year**	**Full reference**	**Country (for empirical studies) / Publication type (for other studies)**
1.	[9]	Ono et al. 2013	T. Ono, G. Lafortune, and M. Schoenstein, "Health workforce planning in OECD countries: a review of 26 projection models from 18 countries," OECD Publishing, 62, 2013. doi: http://dx.doi.org/10.1787/5k44t787zcwb-en.	theoretical, conceptual paper
2.	[13]	Drennan & Ross 2019	V. M. Drennan and F. Ross, "Global nurse shortages-the facts, the impact and action for change.," *Br. Med. Bull.*, vol. 130, no. 1, pp. 25–37, Jun. 2019, doi: 10.1093/bmb/ldz014.	literature review
3.	[14]	Shembavnekar et al. 2022	N. Shembavnekar *et al.*, "NHS workforce projections 2022," 2022.	Report
4.	[15]	WHO 2021	World Health Organization, *Health labour market analysis guidebook*. Geneva, Switzerland: World Health Organization, 2021.	Guidebook
5.	[16]	Schneider 2021	Schneider M, "Health workforce shortage in EU27 in the light of accounting systems," 2021.	theoretical, conceptual paper
6.	[17]	European Comission; SEPEN consortium 2021	S. B. Kovács E., Szegner P., Langner L., Sziklai M., Szócska M., Sermeus W., Van Hoegaerden M., Van Deun E., "Mapping of national health workforce planning and policies in the EU-28," 2021.	Report
7.	[26]	Van Greuningen et al. 2012	M. Van Greuningen, R. S. Batenburg, and L. F. Van der Velden, "Ten years of health workforce planning in the Netherlands: a tentative evaluation of GP planning as an example.," *Hum. Resour. Health*, vol. 10, p. 21, Aug. 2012, doi: 10.1186/1478-4491-10-21.	the Netherlands

8.	[27]	Pandit et al. 2010	J. J. Pandit, A. N. Tavare, and P. Millard, "Why are there local shortfalls in anaesthesia consultant staffing?: A case study of operational workforce planning," *J. Heal. Organ. Manag.*, vol. 24, no. 1, pp. 4–21, 2010, doi: 10.1108/14777261011029543.	UK
9.	[28]	Maier & Afentakis 2013	T. Maier and A. Afentakis, "Forecasting supply and demand in nursing professions: impacts of occupational flexibility and employment structure in Germany.," *Hum. Resour. Health*, vol. 11, p. 24, Jun. 2013, doi: 10.1186/1478-4491-11-24.	Germany
10.	[29]	Tsiouli et al. 2016	K. Tsiouli, K. Karamesinis, G. S. Antonarakis, and P. Christou, "Prediction model of regional orthodontic workforce needs, using Greece as an example," *Eur. J. Paediatr. Dent.*, vol. 17, no. 1, pp. 29–33, 2016, [Online]. Available: https://www.embase.com/search/results?subaction=viewrecord&id=L613678098&from=export	Greece
11.	[30]	Lovkyte et al. 2003	L. Lovkyte, J. Reamy, and Z. Padaiga, "Physicians resources in Lithuania: change comes slowly.," *Croat. Med. J.*, vol. 44, no. 2, pp. 207–213, Apr. 2003.	Lithuania
12.	[31]	Stierli et al. 2021	R. Stierli *et al.*, "Primary Care Physician Workforce 2020 to 2025 - a cross-sectional study for the Canton of Bern.," *Swiss Med. Wkly.*, vol. 151, p. w30024, Aug. 2021, doi: 10.4414/SMW.2021.w30024.	Switzerland
13.	[32]	Barber & López-Valcárcel 2010	P. Barber and B. G. López-Valcárcel, "Forecasting the need for medical specialists in Spain: application of a system dynamics model.," *Hum. Resour. Health*, vol. 8, p. 24, Oct. 2010, doi: 10.1186/1478-4491-8-24.	Spain
14.	[33]	Grech V. et al. 2012	V. Grech, M. Cassar, and S. Distefano, "Nurse staffing levels on the NPICU in the island of Malta.," *J. Pediatr. intensive care*, vol. 1, no. 1, pp. 25–29, Mar. 2012, doi: 10.3233/PIC-2012-005.	Malta
15.	[34]	Papp et. al 2019	M. Papp, L. Korosi, J. Sandor, C. Nagy, A. Juhasz, and R. Adany, "Workforce crisis in primary healthcare worldwide: Hungarian example in a longitudinal follow-up study," *BMJ Open*, vol. 9, no. 7, 2019, doi: 10.1136/bmjopen-2018-024957	Hungary

16.	[35]	Liu et al. 2017	J. X. Liu, Y. Goryakin, A. Maeda, T. Bruckner, and R. Scheffler, "Global Health Workforce Labor Market Projections for 2030.," *Hum. Resour. Health*, vol. 15, no. 1, p. 11, Feb. 2017, doi: 10.1186/s12960-017-0187-2.	Multicountry - 165 countries
17.	[36]	Scheffler et al. 2008	R. M. Scheffler, J. X. Liu, Y. Kinfu, and M. R. Dal Poz, "Forecasting the global shortage of physicians: an economic- and needs-based approach.," *Bull. World Health Organ.*, vol. 86, no. 7, pp. 516-523B, Jul. 2008, doi: 10.2471/blt.07.046474.	Multicountry - 158 countries
18.	[37]	Scheffler et al. 2018	R. M. Scheffler *et al.*, "Forecasting imbalances in the global health labor market and devising policy responses.," *Hum. Resour. Health*, vol. 16, no. 1, p. 5, Jan. 2018, doi: 10.1186/s12960-017-0264-6.	Multicountry
19.	[38]	Taylor et al. 2018	C. Taylor, I. C. McManus, and I. Davison, "Would changing the selection process for GP trainees stem the workforce crisis? A cohort study using multiple-imputation and simulation," *BMC Med. Educ.*, vol. 18, no. 1, p. 81, Apr. 2018, doi: 10.1186/s12909-018-1160-z.	UK
20.	[39]	Lodi et al. 2015	A. Lodi, P. Tubertini, R. Grilli, A. Mazzocchetti, C. Ruozi, and F. Senese, "Needs forecast and fund allocation of medical specialty positions in Emilia-Romagna (Italy) by system dynamics and integer programming," *Heal. Syst.*, vol. 5, no. 3, pp. 213–236, 2016.	Italy
21.	[40]	Gialama et al. 2019	F. Gialama, M. Saridi, P. Prezerakos, Y. Pollalis, X. Contiades, and K. Souliotis, "The implementation process of the Workload Indicators Staffing Need (WISN) method by WHO in determining midwifery staff requirements in Greek Hospitals.," *Eur. J. midwifery*, vol. 3, p. 1, 2019, doi: 10.18332/ejm/100559.	Greece
22.	[41]	Van Greuningen et al. 2016	M. Van Greuningen, "Health workforce planning in the Netherlands," *Utr. Tilbg. Univ.*, 2016.	the Netherlands
23.	[42]	Gallagher et al. 2010	J. E. Gallagher, E. R. Kleinman, and P. R. Harper, "Modelling workforce skill-mix: how can dental professionals meet the needs and demands of older people in England?," *Br. Dent. J.*, vol. 208, no. 3, pp. E6–E6, 2010.	UK

24.	[43]	Starkiene et al. 2005	L. Starkiene, K. Smigelskas, Z. Padaiga, and J. Reamy, "The future prospects of Lithuanian family physicians: a 10-year forecasting study.," *BMC Fam. Pract.*, vol. 6, p. 41, Oct. 2005, doi: 10.1186/1471-2296-6-41.	Lithuania
25.	[44]	Ravindran et al. 2021	S. Ravindran *et al.*, "Bowel cancer screening workforce survey: developing the endoscopy workforce for 2025 and beyond," *Frontline Gastroenterol.*, vol. 13, no. 1, pp. 12–19, 2022, doi: 10.1136/flgastro-2021-101790.	UK
26.	[45]	Teljeur et al. 2010	C. Teljeur, S. Thomas, F. D. O'Kelly, and T. O'Dowd, "General practitioner workforce planning: assessment of four policy directions.," *BMC Health Serv. Res.*, vol. 10, p. 148, Jun. 2010, doi: 10.1186/1472-6963-10-148.	UK
27.	[46]	Harkin et al. 2016	D. W. Harkin, J. D. Beard, C. P. Shearman, M. G. Wyatt, and R. C. Surg, "Predicted shortage of vascular surgeons in the United Kingdom: A matter for debate?," *Surg. R. Coll. Surg. EDINBURGH Irel.*, vol. 14, no. 5, pp. 245–251, Oct. 2016, doi: 10.1016/j.surge.2015.10.004	UK
28.	[47]	Hegarty et al. 2022	H. Hegarty *et al.*, "Nurse staffing levels within acute care: results of a national day of care survey.," *BMC Health Serv. Res.*, vol. 22, no. 1, p. 493, Apr. 2022, doi: 10.1186/s12913-022-07562-w.	UK
29.	[48]	Maresova et al. 2020	P. Maresova, M. Prochazka, S. Barakovic, J. Baraković Husić, and K. Kuca, "A Shortage in the Number of Nurses-A Case Study from a Selected Region in the Czech Republic and International Context.," *Healthc. (Basel, Switzerland)*, vol. 8, no. 2, Jun. 2020, doi: 10.3390/healthcare8020152.	Czech Republic
30.	[49]	Tsai et al. 2012	T.-C. Tsai, M. Eliasziw, and D.-F. Chen, "Predicting the demand of physician workforce: an international model based on 'crowd behaviors'.," *BMC Health Serv. Res.*, vol. 12, p. 79, Mar. 2012, doi: 10.1186/1472-6963-12-79.	Multicountry - 130 countries

31.	[50]	Boniol et al. 2022	M. Boniol, T. Kunjumen, T. S. Nair, A. Siyam, J. Campbell, and K. Diallo, “The global health workforce stock and distribution in 2020 and 2030: a threat to equity and ‘universal’ health coverage?,” *BMJ Glob. Heal.*, vol. 7, no. 6, Jun. 2022, doi: 10.1136/bmjgh-2022-009316 WE - Science Citation Index Expanded (SCI-EXPANDED) WE - Social Science Citation Index (SSCI).	Multicountry
32.	[51]	Dussault et al. 2010	G. Dussault, J. Buchan, W. Sermeus, and Z. Padaiga, “Assessing future health workforce needs,” World Health Organization Copenhagen, 2010.	Report
33.	[52]	Mara 2020	I. Mara, “Health professionals wanted: Chain mobility across European countries,” wiiw Research Report, 2020.	Report
34.	[53]	Roberfroid et al. 2008	D. Roberfroid, S. Stordeur, C. Camberlin, C. Van de Voorde, F. Vrijens, and C. Leonard, “Physician workforce supply in Belgium: current situation and challenges,” Belgian Health Care Knowledge Centre, 2008.	Report
35.	[54]	WHO 2010	World Health Organization, “Models and tools for health workforce planning and projections,” World Health Organization, Geneva, Switzerland, 2010.	theoretical, conceptual paper
36.	[55]	Kroezen et al. 2017	M. Kroezen, M. Van Hoegaerden, and R. Batenburg, “The Joint Action on Health Workforce Planning and Forecasting: Results of a European programme to improve health workforce policies,” *Health Policy (New. York).*, vol. 122, no. 2, pp. 87–93, 2018.	theoretical, conceptual paper
37.	[56]	Simoens et al. 2005	S. Simoens, M. Villeneuve, and J. Hurst, “Tackling nurse shortages in OECD countries,” OECD, 2005.	theoretical, conceptual paper

38.	[57]	Malgieri et al. 2015	A. Malgieri, P. Michelutti, and M. Van Hoegaerden, “Handbook on health workforce planning methodologies across EU countries,” *Bratislava Minist. Heal. Slovak Repub.*, 2015.	theoretical, conceptual paper
